# Longitudinal CT-based deep learning radiomics for predicting prognosis in esophageal squamous cell carcinoma treated with definitive chemoradiotherapy: a two-center study

**DOI:** 10.7150/jca.126804

**Published:** 2026-03-30

**Authors:** Xinyang Hu, Wubulaishan Maitudi, Xi Chen, Lina Zhao, Qingsong Pang

**Affiliations:** 1 Department of Radiation Oncology, Tianjin Medical University Cancer Institute & Hospital, National Clinical Research Center for Cancer, Tianjin's Clinical Research Center for Cancer, Tianjin, China.; 2 Tianjin Key Laboratory of Digestive Cancer, Tianjin, China.; 3 State Key Laboratory of Druggability Evaluation and Systematic Translational Medicine, Tianjin, China.; 4 Hetian District People's Hospital, Hetian, China.; 5 Department of Radiation Oncology, Xijing Hospital, Air Force Medical University, Xi'an, China.

**Keywords:** esophageal squamous cell carcinoma, deep learning, radiomics, overall survival

## Abstract

**Purpose:**

To develop and validate a longitudinal deep-learning based survival prediction model for esophageal squamous cell carcinoma (ESCC) patients who received definitive concurrent chemoradiotherapy (CCRT).

**Methods:**

A total of 257 ESCC patients from two centers were recruited. Among them, 205 patients were in the training cohort and 52 in the external testing cohort. The CrossFormer algorithm was utilized to extract features from pre- and post- treatment CECT scans. We constructed clinical, Delta-radiomics and deep-learning models. Models were evaluated by the C-index and integrated Brier score (iBS). Prognostic stratification was performed based on risk scores, and model interpretability was evaluated using Grad-CAM and SurvSHAP(t).

**Results:**

The Fusion model demonstrated superior predictive performance, achieving a C-index of 0.768 (95% CI: 0.731-0.804) in the training cohort and 0.734 (95% CI:0.665-0.803) in the testing cohort. The Fusion model also showed the best calibration (Training cohort: iBS=0.096; Testing cohort: iBS=0.151). Patients were stratified into high-risk and low-risk groups based on risk scores, with significant differences in overall survival (OS) between the groups (*P* < 0.001).

**Conclusion:**

We developed a model integrating longitudinal CECT scans to predict OS in ESCC patients undergoing CCRT. The results highlight the importance of capturing tumor changes during treatment for accurate prognostic stratification. The model shows its potential for guiding personalized treatment strategies in clinical practice.

## Introduction

Esophageal carcinoma is one of the most common malignant tumors, ranking sixth in cancer-related mortality, with over 50% of cases originating from China 1. In China, esophageal squamous cell carcinoma (ESCC) represents over 90% of all esophageal cancer diagnoses 2. Due to the lack of typical symptoms in the early stages, patients are often diagnosed at advanced stages, leading to a relatively poor prognosis 3.

Curative surgery is feasible for only a limited subset of patients diagnosed with ESCC. For those with advanced esophageal cancer and those unwilling to undergo surgery, the current standard treatment regimen is definitive concurrent chemoradiotherapy (CCRT) 4. Compared to surgical treatment, CCRT results in poorer survival outcomes but is superior in health-related quality of life. In recent decades, radiotherapy technology has made significant advancements. However, the 5-year overall survival rate for ESCC patients receiving CCRT remains unsatisfactory 5. To better develop personalized treatment strategies, there is a need for improved predictive methods to identify ESCC patients who can benefit from CCRT.

Radiomics, a rapidly evolving predictive approach, utilizes high-throughput feature extraction from multimodality medical images to characterize tumors quantitatively. Its application in ESCC has been explored extensively, with studies focusing on clinical staging, metastasis prediction, treatment response evaluation, and outcomes following CCRT 6-8. However, traditional radiomics requires precise tumor segmentation and is limited in the amount of information that can be extracted from images.

Deep learning, as an emerging machine learning technique, differs from traditional machine learning approaches in that it can directly process raw data and automatically develop the necessary representations for pattern recognition, thereby extracting more information from images. Numerous studies have shown that deep learning models achieve significantly better performance than traditional methods 9-11. Recent deep learning models often use single-phase pretreatment scans to predict response to CCRT, while ignoring changes in the tumor during treatment or follow-up 12,13. Some studies have attempted to integrate images from both pre- and post-treatment for outcome prediction, and these models have shown significantly better performance compared to single-time-point models 14,15. To our knowledge, no studies have yet applied deep learning methods integrating longitudinal CT scans for the prognostic evaluation of ESCC patients undergoing CCRT. Additionally, more advanced techniques are not used.

This study aims to develop and validate a deep learning model based on CrossFormer for predicting overall survival (OS) in ESCC patients treated with CCRT, utilizing longitudinal contrast-enhanced CT (CECT) images. The model can predict survival risk at different time points and has the potential to guide personalized treatment strategies.

## Methods

### Patient selection and follow-up

**Figure 1:**
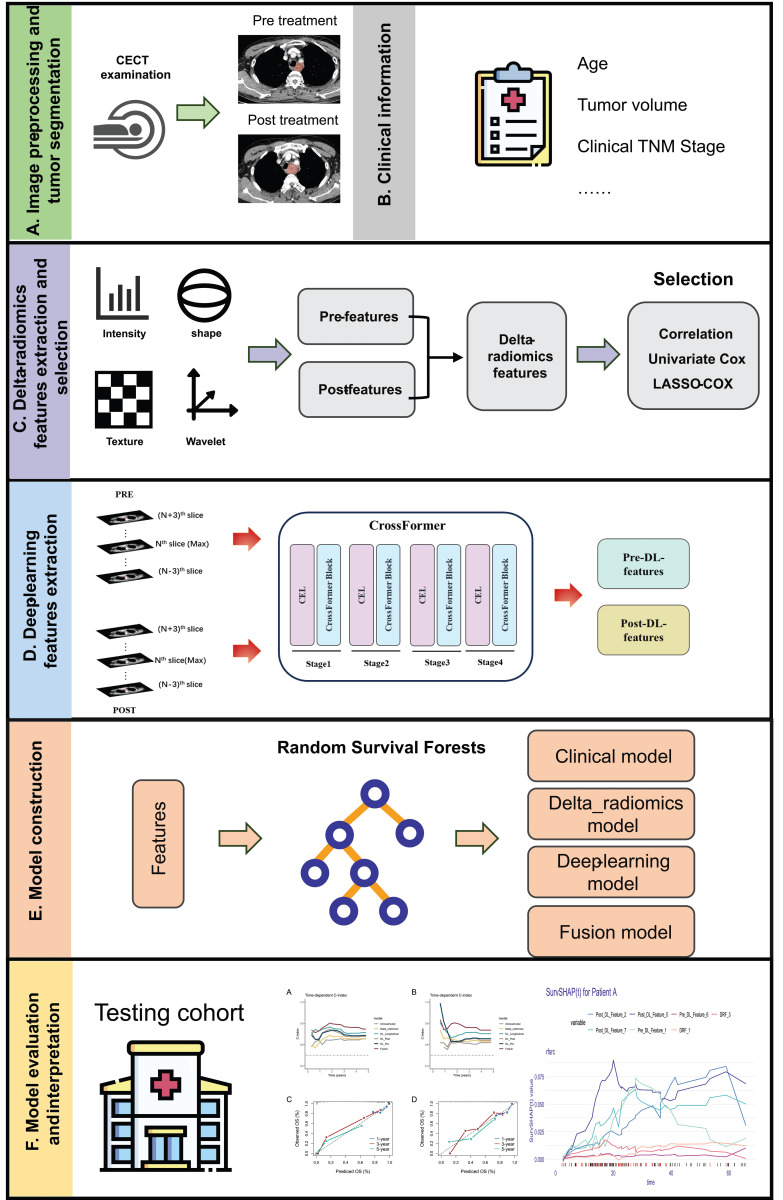
Workflow of this study.

Workflow of this study is depicted in Figure 1. This study recruited ESCC patients who received CCRT at Tianjin Medical University Cancer Institute & Hospital between January 2018 and December 2022. These patients were used to develop machine learning models. Additionally, a testing cohort was retrospectively enrolled from Xijing Hospital, consisting of patients from September 2019 to December 2020. Patients included in this study met the following criteria: (1) age ≥ 18; (2) histologically confirmed ESCC; (3) cT_3_-_4_N_0_M_0_/cT_1-4_N_+_M_0_ or cM_1_ (only including positive nonregional lymph nodes) according to the AJCC 8th edition; (4) treated with intensity-modulated radiation therapy (IMRT) with total radiation doses ≥ 50 Gy using conventional fractionated radiotherapy; (5) received at least one cycle of chemotherapy during radiotherapy; and (6) availability of two CECT scans, one at baseline and another within 1 month after CCRT. The exclusion criteria were as follows: (1) poor image quality or incomplete medical records; and (2) patients who underwent radical surgical treatment. Post-treatment follow-up evaluations were conducted at 3-month intervals during the initial 2-year period, followed by 6-month intervals thereafter. OS was defined as the duration from the start date of radiotherapy to death, with data for living patients censored at their last follow-up. This study was approved by the ethics committee of Tianjin Medical University Cancer Institute and Hospital (No. bc20250345). Given the retrospective nature of the research, informed consent was waived.

### Treatment

Patients received CCRT, wherein radiotherapy was IMRT. A total radiation dose of 50-60 Gy was delivered with 6-15 MV X-rays, with 1.8 or 2 Gy per fraction and 5 fractions per week. Patients received at least one course of concurrent chemotherapy with the following regimens: (1) cisplatin plus 5-fluorouracil; or (2) docetaxel or paclitaxel plus cisplatin. The choice of regimen was individualized for each patient, taking into account their age, overall health status, and the clinical discretion exercised by the physician.

### CT image acquisition and processing

The details of CT image acquisition parameters are provided in Table S1. All original intravenous CECT images underwent image preprocessing, included adjusting the Hounsfield units of CT images to the standard window (window center at 50 and window width at 350), resampling voxels to 1×1×1 mm and intensity normalization using the z-score method.

### The region of interest

The region of interest (ROI) encompassed the entire gross tumor volume (GTV), defined slice by slice on mediastinal window settings. Two experienced radiation physicians, each with over 5 years in the field, delineated the pre- and post-treatment ROIs by using the 3D Slicer software (version 5.4.0, www.slicer.org) 16. And one radiologist with 30 years of experience reexamined all ROIs.

### Hand-craft delta-radiomics features extraction and selection

PyRadiomics package (https://pyradiomics.readthedocs.io/en/latest/features.html) was used to extracted features from each ROI, and features with an intraclass correlation coefficient (ICC) of ≥ 0.8 were retained for subsequent analysis. We used pearson correlation analysis to calculate the correlation coefficients between variables to identify all variable pairs. For each pair of highly correlated variables with a correlation coefficient greater than 0.8, only one was retained. The Delta-radiomics features (DRFs) were calculated as the RF_Post_ value minus the RF_Pre_ value, divided by the RF_Pre_ value:



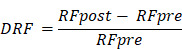



Univariate Cox regression was then employed for the preliminary screening of survival-associated features (*P* < 0.1). Finally, the least absolute shrinkage and selection operator (LASSO) Cox regression was applied to identify and retain DRFs with non-zero coefficients which were used for the modeling.

### Deep-learning features extraction

For each patient, the images before and after treatment were cropped using masks of ROIs, and the largest cross-sectional slice along with three slices above and below it was selected. The 7 slices obtained from the pre-CT or post-CT were regarded as 7 channels. The minimal rectangle that could encompass the tumor area across all slices was treated as ROI for the input. Then, the ROIs cropped from slices were resized to 224×224 using nearest-neighbor interpolation. Since the model uses multiple 2D images, occupying a middle ground between 2D and 3D, it is referred to as a 2.5D model.

We employed a foundational model called CrossFormer to extract features 17. The model is designed to handle variable-sized inputs through a Cross-scale Embedding Layer (CEL) and a Long Short Distance Attention (LSDA) mechanism. The CEL combines each embedding with multi-scale patches, enabling the self-attention module to capture cross-scale features effectively. Meanwhile, the LSDA separates the self-attention mechanism into short-distance and long-distance components. This design not only enhances computational efficiency but also ensures the retention of both fine-grained and coarse-grained features in the embeddings. For detailed information, the project can be accessed on GitHub (https://github.com/cheerss/CrossFormer). All experiments were conducted on an RTX 4080 Super GPU. The Adam optimizer was employed to update the model parameters, with a batch size set to 32. In addition, Dropout regularization was introduced to prevent overfitting. To enlarge the dataset and enhance the robustness of the model, random horizontal flipping and random vertical flipping were employed.

Pre-CT-Features and Post-CT-Features were extracted from the average pooling layer of CrossFormer based on the multichannel pictures. Each feature set was characterized by a vector containing 512 features. To better understand the key feature locations identified by CrossFormer, visual heatmaps were generated using Gradient-weighted Class Activation Mapping (Grad-CAM) 18.

### Model building and interpretation

Random Survival Forest (RSF) is an ensemble method where each tree consists of nodes that are split based on the variable maximizing the survival difference between the child nodes 19. In this study, the log-rank test was used as the splitting criterion. We utilized selected DRFs and dimensionality-reduced features to construct 4 models, named Delta-radiomics model, DL_Pre model, DL_Post model and DL_Longitudinal model, using RSF algorithm. In addition, a clinical model was constructed based on age, clinical stage, and tumor volume reduction rate. In the Fusion model, we concatenated all selected feature sets including clinical, delta-radiomics and deep-learning features into a single feature matrix before modeling with the RSF algorithm. RSF models was constructed using the randomForestSRC package in R. The forest consisted of 400 trees, with a terminal node size of 10 and 4 variables randomly selected at each split. Risk scores for each patient were then calculated based on the predictions from these models. The C-index was used to evaluate the models' discrimination ability. Additionally, the integrated Brier score (iBS) was applied to assess calibration ability. The 95% confidence intervals (CIs) were generated through bootstrap resampling, performed 1000 times. The survex R package was used to interpret the RSF model 20. SurvSHAP(t), a specialized extension of Shapley additive explanations (SHAP) for survival models 21, was applied to evaluate the contribution of each predictor variable to the model's survival function. Based on the median risk score, patients are divided into high-risk and low-risk groups. Kaplan-Meier survival analysis was performed to assess the difference in prognosis between groups.

### Statistical analysis

All statistical analyses were performed using R (version 4.2.3; www.Rproject.org) and Python software (version 3.8.0). Categorical variables were analyzed using chi-squared test or Fisher exact test, while continuous variables were assessed with the Mann-Whitney U test or independent samples t-test. The statistical significance of the difference in C-index between models was evaluated using the survcomp R package. A two-sided *P*-value < 0.05 was considered statistically significant. All feature selection and model training processes were conducted using only the training cohort data, while the test cohort was used for model evaluation. The optimal hyperparameters of the models were determined based on five-fold cross-validation.

## Results

### Patients

A total of 257 patients were finally recruited from two centers. Table 1 lists the detailed clinicopathological information of the patients in the training (n = 205) and external testing cohorts (n = 52). In the training cohort, the median volumes for GTV_pre_ and GTV_post_ were 26.4 cm³ and 19.5 cm³ respectively, which were lower than those in the testing cohort (32.5 cm³ and 21.1 cm³). Patients in the external testing cohort had relatively earlier clinical stages (*P* < 0.001). The median follow-up time was 48.5 months.

### Analysis of delta-radiomics and DL features

A total of 834 radiomics features were extracted, consisting of 105 original features and 728 wavelet-transformed features. Based on feature classes, these could be further categorized into 4 shape-based features, 162 first-order features, 198 gray-level co-occurrence matrix (GLCM) features, 126 gray-level dependence matrix (GLDM) features, 144 gray-level run-length matrix (GLRLM) features, 144 gray-level size-zone matrix (GLSZM) features, and 45 neighboring gray-tone difference matrix (NGTDM) features. After correlation analysis and univariate Cox regression, 106 DRFs were selected. LASSO-Cox regression ultimately selected 10 features with non-zero coefficients. Table S2 presented the chosen DRFs and their coefficients.

To prevent model overfitting, we used principal component analysis (PCA) to reduce the dimensionality of DL-features, ultimately retaining 10 key features for each set. To assess the correlation between Pre-CT-Features and Post-CT-Features, we employed the Spearman method for correlation analysis. As shown in Figure S1, the inter-correlation among DL-features within each cohort is extremely low.

### Development and performance of models

**Figure 2:**
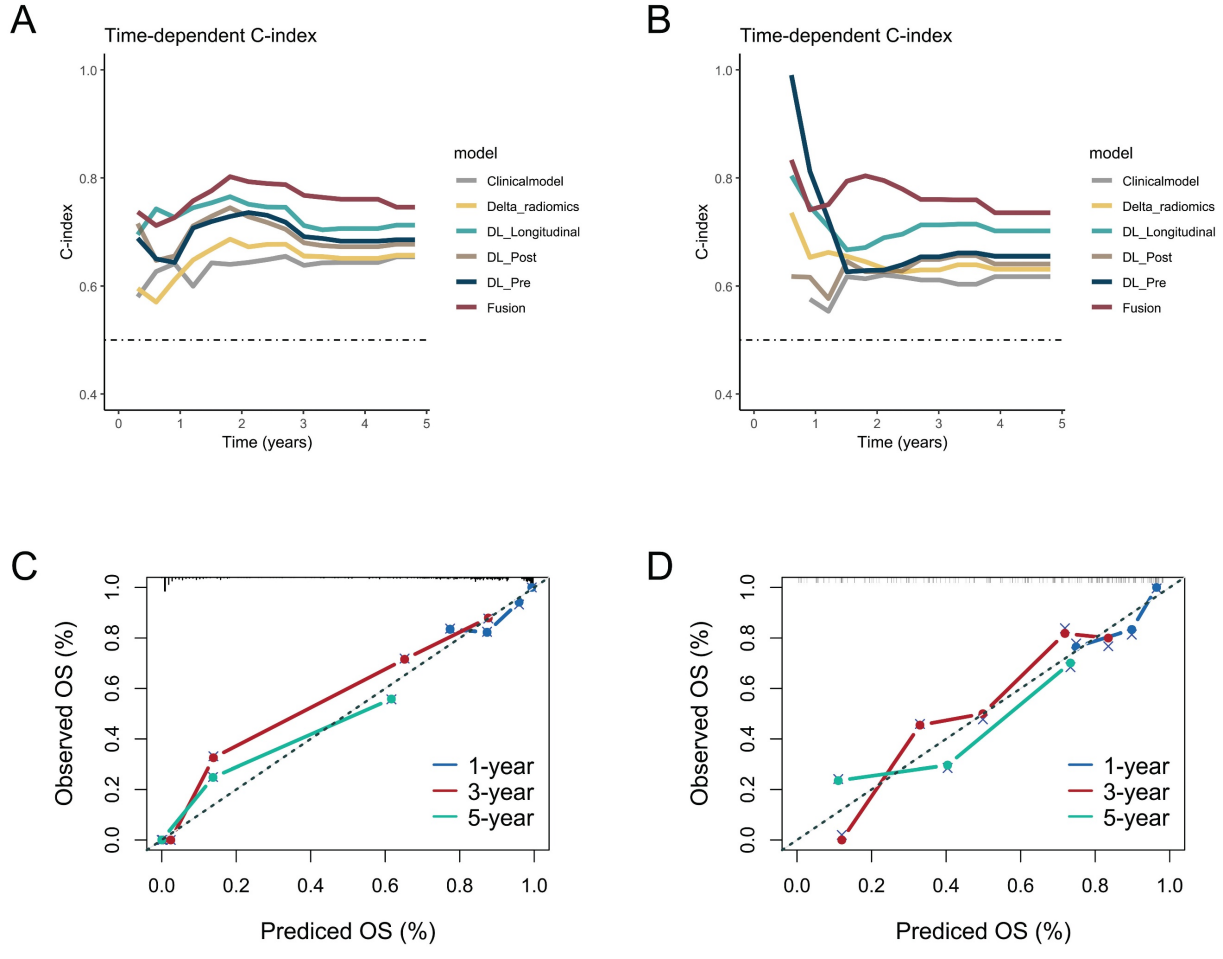
The time-dependent C-index curves of different model in the (A) training cohort and (B) testing cohort. The calibration curves for the Fusion model in the (C) training cohort and (D) testing cohort.

We also built a Fusion model by incorporating selected clinical, DRFS, Pre-CT-Features and Post-CT-Features. The performance metrics of the models across research cohorts are summarized in Table 2. In the training cohort, the Fusion model demonstrated the highest discriminative ability, with a C-index of 0.768 (95% CI: 0.731-0.804), which was significantly superior to the Clinical model (*P* < 0.001). The DL_Longitudinal model also performed well (C-index = 0.732, *P* = 0.002). Regarding calibration, the Fusion model achieved the lowest iBS, indicating the best overall predictive accuracy. In the testing cohort, only the Fusion model showed a significant advantage compared to the Clinical model (C-index: 0.734 vs. 0.619, *P* = 0.021). All deep learning-based models and the Fusion model significantly improved calibration over the Clinical model (*P* < 0.001). Overall, the Fusion model consistently outperformed other models in both discrimination and calibration across cohorts, highlighting its robustness and potential for clinical application. The curves for time-dependent C-index and the calibration curves for the Fusion model were shown in Figure 2.

### Prognostic stratification

**Figure 3:**
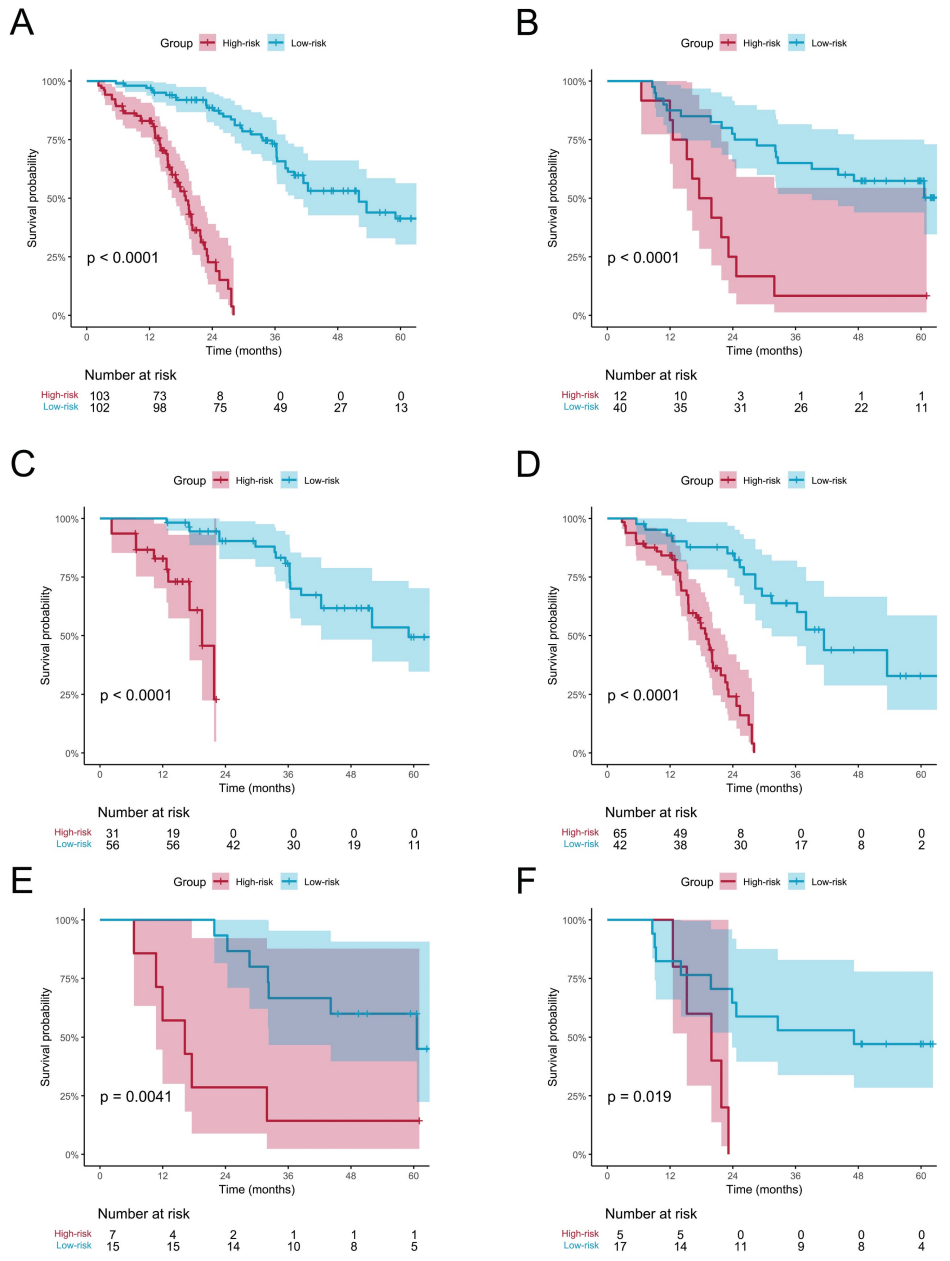
Kaplan-Meier analysis of overall survival (OS) based on the risk groups stratified by the Fusion model. OS curves for the high- and low-risk groups in the (A) training and (B) testing cohorts. Subgroup analysis of OS among patients with clinical stage III and IV in the (C, D) training and (E, F) testing cohorts.

Patients were classified into high-risk and low-risk groups based on risk scores computed by the Fusion model. The KM curve showed a significant difference in OS between patients in the low-risk group and high-risk group. The Log-rank test indicated that in both the training and testing cohort, the overall survival (OS) could be significantly stratified according to the riskscore from the Fusion model (Figure 3A-B). Additionally, we performed Kaplan-Meier survival analysis for OS based on risk groups among patients with clinical stage III and IV. The analysis revealed significant survival differences across all groups (Figure 3C-F). Subsequent multivariate Cox regression analysis, adjusted for clinicopathologic factors, demonstrated that the risk score served as a robust and independent prognostic factor for OS (HR = 7.33, *P* < 0.001, Table S3).

### Model interpretation

**Figure 4:**
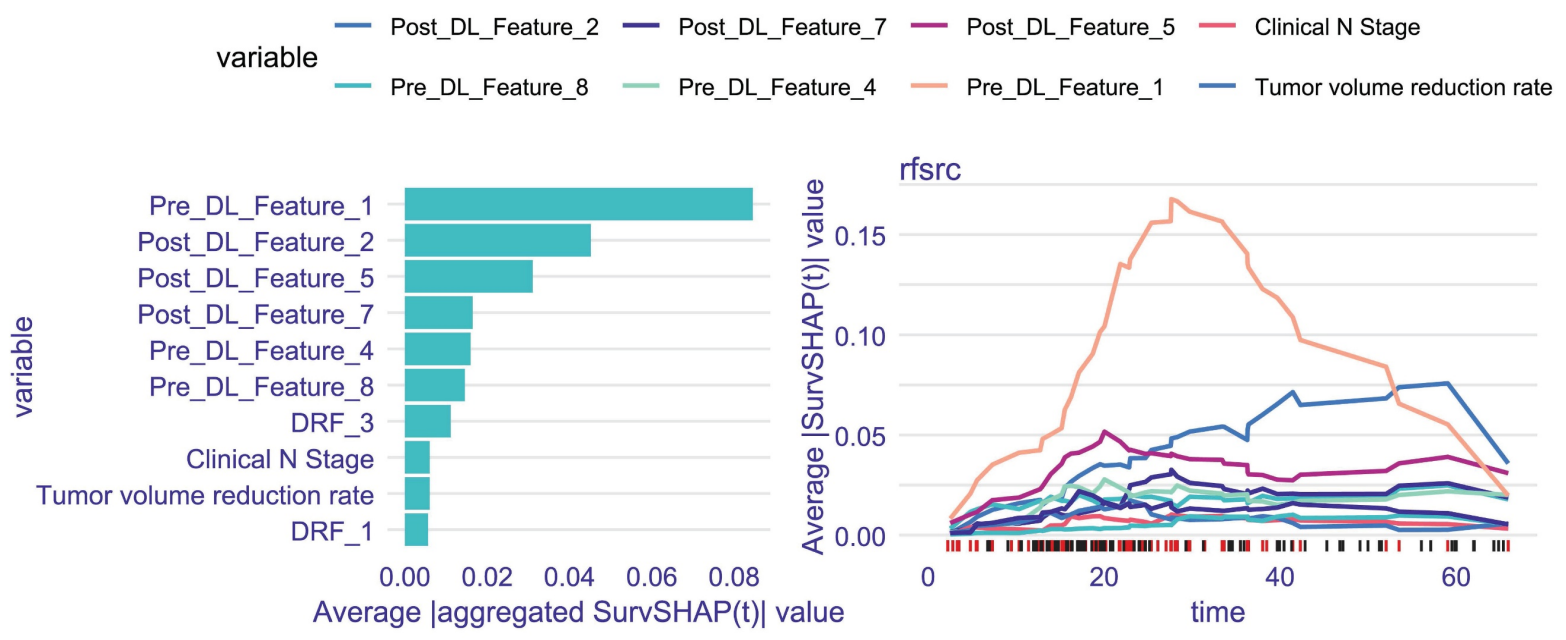
The left panel depicts top10 most important variables identified by the average absolute aggregated SurvSHAP(t) value; The right of panel depicts Change in the impact of each variable.

Figure 4 illustrates the ranking of the most significant variables based on their average absolute aggregated SurvSHAP(t) values within the Fusion-model. The right panel of Figure 4 depicts how the average absolute SurvSHAP(t) values of these variables change throughout the follow-up period. Higher values for features indicate a greater impact on OS. Pre_DL_Feature_1 exhibited the highest level of impact.

**Figure 5:**
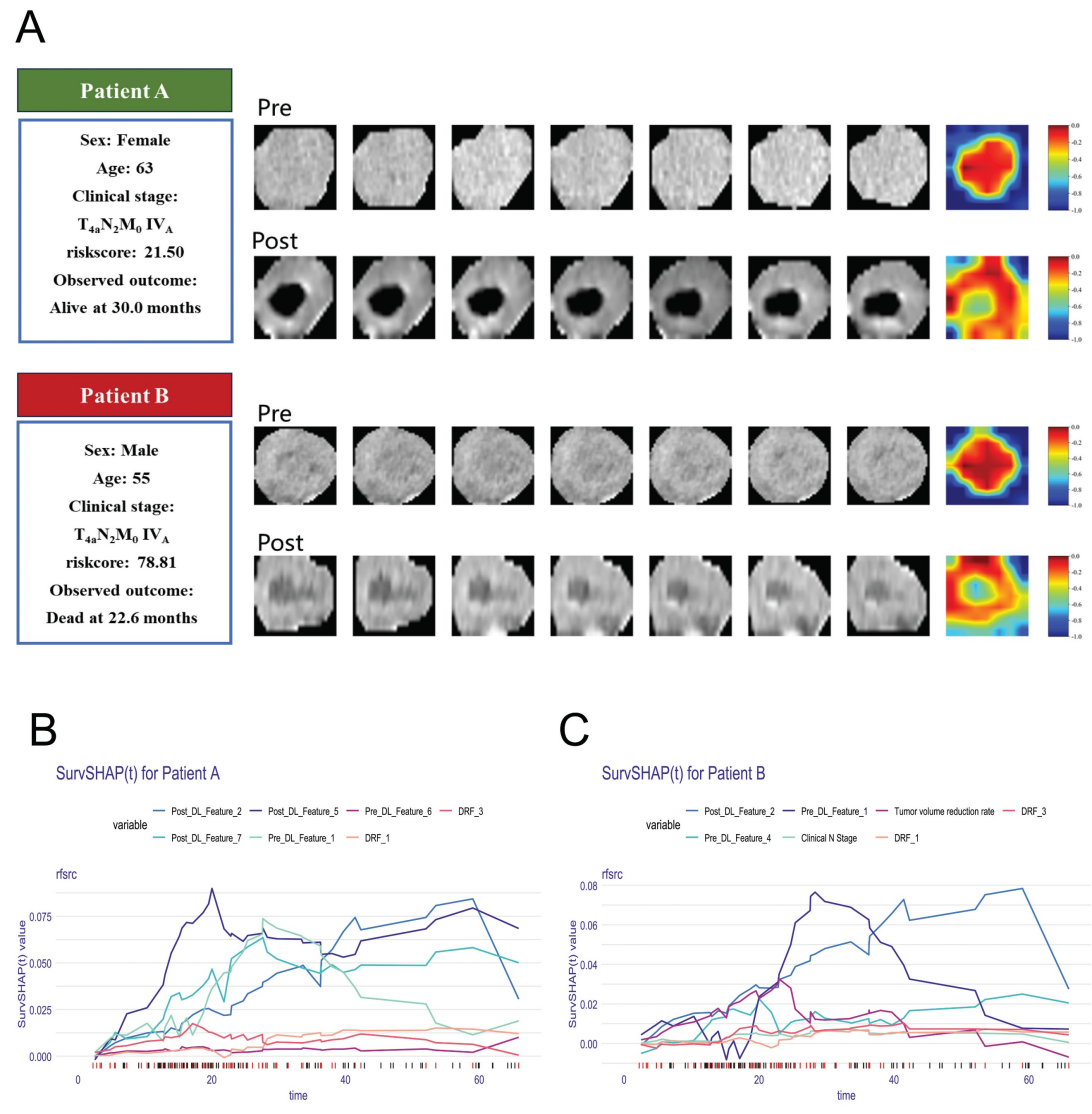
(A) Clinical information and Grad-CAM visualizations of representative patients. The darker red areas mean more contribution to prediction. B-C explain the prediction of patients with SurvSHAP(t).

Figure 5 provides some representative patients. Although the two patients were diagnosed at the identical clinical stage, their prognostic outcomes diverged significantly. The model demonstrated a strong ability to make this critical distinction. The Grad-CAM heatmaps indicate that key deep-learning features are extracted from center regions of the tumor. By calculating the SurvSHAP(t) values, the model's prediction results for individual patients were decomposed into the independent contributions of each feature, thereby intuitively illustrating how different features influence OS prediction over time (Figure 5 B-C).

## Discussion

To the best of our knowledge, this is the first study to apply deep learning algorithms for feature extraction from longitudinal CT scans to predict survival outcomes in ESCC patients treated with CCRT. Our results demonstrate that integrating pre- and post-treatment imaging data significantly improves prognostic accuracy compared with single-time-point models, emphasizing the importance of longitudinal tumor characterization.

In our study, the 1-year, 2-year, 3-year and 5-year OS rates were 89.3%, 64.1%, 49.5%, and 33.7%, respectively, in keeping with rates reported in previous study 5. The absence of a gold standard for evaluating tumor treatment efficacy presents significant challenges in achieving accurate prognostic stratification for patients with ESCC. Previous radiomics studies have explored the prognostic potential of imaging features in ESCC. For instance, Li *et al.* combined radiomics features with clinical variables to predict chemoradiotherapy response and 2-year OS, achieving AUCs of 0.71 and 0.70 in training and validation cohorts 22. And Cui *et al.* developed a pre-treatment CECT radiomics model predicting 3-year OS with AUCs exceeding 0.83 6. Kawahara *et al.* further proposed a nomogram integrating CT, PET, and dosiomics features, achieving a C-index above 0.90 in external validation 12. These studies demonstrate that quantitative imaging biomarkers hold promise for ESCC prognosis. However, most studies were single-center and based only on pre-treatment scans, neglecting dynamic tumor changes after therapy. Recent delta-radiomics study have shown that time-dependent imaging features significantly enhance prognostic prediction in ESCC patients undergoing CCRT (C-index = 0.821, 95% CI: 0.735-0.907) 23. Similarly, our longitudinal approach captures post-treatment evolution, offering a more comprehensive assessment of treatment response and survival.

Our study has several notable advantages compared to previous studies. First, we employed deep learning algorithms to extract features from multi-dimensional images, enabling the extraction of more comprehensive tumor information from CECT scans compared to conventional clinical and radiologic characteristics 14,24. Second, we collected longitudinal CECT data, which captured tumor changes during CCRT. The Fusion-model, integrating information from both pre- and post-treatment data, outperformed models using single-time-point data. In the testing cohort, the Fusion-model achieved the highest C-index and the lowest iBS value. These results align with findings from studies that utilized longitudinal imaging to predict pathological complete response (pCR) 15,25. Moreover, the model was validated in an external testing set.

Our study found that assessing patients' prognosis based on the degree of tumor volume regression cannot accurately predict outcomes, which may be related to tumor heterogeneity. Grad-CAM visualizations revealed that key predictive features were primarily concentrated in central tumor regions. By integrating global and local explanation methods, we quantified the contribution of individual features to patient-specific survival predictions, thereby enhancing interpretability. For example, Patients A and B had similar clinical stages but exhibited significantly different risk scores. Patient A, who had a lower risk score of 21.50, alive at 30 months, while Patient B, with a risk score of 78.81, died at 22.6 months. An increasing number of studies are exploring the optimal approaches for maintenance therapy following CCRT in ESCC 26. Accurate prognostic stratification of patients may help identify those suitable for adjuvant therapy and reduce overtreatment.

While our study constructed a highly accurate survival prediction model using deep learning techniques to precisely stratify ESCC patients undergoing CCRT, it is important to acknowledge some limitations. First, this study is retrospective, and baseline differences in age, clinical stage, and metastatic status were observed between the training and external validation cohorts. Future work should include larger multicenter datasets to further validate model robustness and mitigate potential bias introduced by baseline heterogeneity. Second, some patients were excluded because they did not follow up at our hospital after treatment or underwent non-contrast CT scans during follow-up, which may have introduced patient selection bias. Finally, we did not investigate the underlying biological mechanisms potentially associated with prognostic stratification. To deepen our understanding of the prognostic radiomics signature in ESCC and promote its clinical application, future research will explore the intricate interplay among radiomics, genomics, and clinical-pathological phenotypes.

## Conclusion

This study developed a deep learning model integrating longitudinal CECT scans to predict OS in ESCC patients undergoing CCRT. The Fusion-model, which combined pre- and post-treatment imaging data, outperformed single-time-point models, highlighting the importance of capturing tumor changes during treatment for accurate prognostic stratification. The model's interpretability and external validation support its potential for guiding personalized treatment strategies in clinical practice.

## Supplementary Material

Supplementary figures and tables.

## Figures and Tables

**Table 1:** Clinicopathological characteristics of patients.

Characteristics	Total (*n*=257)	Group		*P*
		Training cohort (*n*=205)	Testing cohort (*n*=52)	
Age, Mean±SD	63.1±9.0	61.6±8.1	69.2±9.7	<0.001
Sex, *n* (%)				0.315
Male	234 (91.1)	189 (92.2)	45 (86.5)	
Female	23 (8.9)	16 (7.8)	7 (13.5)	
Tumor Location, *n* (%)				0.639
Proximal third	104 (40.5)	80 (39.0)	24 (46.2)	
Middle third	113 (44.0)	92 (44.9)	21 (40.4)	
Distal third	40 (15.6)	33 (16.1)	7 (13.5)	
Clinical T Stage, *n* (%)				0.001
T1	3 (1.2)	2 (1.0)	1 (1.9)	
T2	9 (3.5)	3 (1.5)	6 (11.5)	
T3	152 (59.1)	129 (62.9)	23 (44.2)	
T4a	59 (23.0)	49 (23.9)	10 (19.2)	
T4b	34 (13.2)	22 (10.7)	12 (23.1)	
Clinical N Stage, *n* (%)				<0.001
N0	19 (7.4)	13 (6.3)	6 (11.5)	
N1	87 (33.9)	59 (28.8)	28 (53.8)	
N2	113 (44.0)	96 (46.8)	17 (32.7)	
N3	38 (14.8)	37 (18.0)	1 (1.9)	
Clinical M Stage, *n* (%)				0.008
M0	232 (90.3)	180 (87.8)	52 (100.0)	
M1	25 (9.7)	25 (12.2)	0 (0.0)	
ClinicalStage, *n* (%)				0.004
II	19 (7.4)	11 (5.4)	8 (15.4)	
III	109 (42.4)	87 (42.4)	22 (42.3)	
IVA	105 (40.9)	83 (40.5)	22 (42.3)	
IVB	24 (9.3)	24 (11.7)	0 (0.0)	
GTV_pre_(cm3), M (Q_1_, Q_3_)	27.4 (17.0, 43.8)	26.4 (15.9, 41.7)	32.5 (23.1, 50.4)	0.010
GTV_post_(cm3), M (Q_1_, Q_3_)	19.9 (12.7, 28.5)	19.5 (12.5, 28.3)	21.1 (15.9, 29.7)	0.235

**Table 2:** Performances of the predictive models in the study cohorts.

	C-index (95%CI)	*P*-value	iBS (95%CI)	*P*-value
Training cohort				
Clinical	0.643 (0.587, 0.699)	-	0.143 (0.118,0.162)	-
Delta_radiomics	0.660 (0.607, 0.714)	0.296	0.140 (0.111,0.155)	< 0.001
DL_Pre	0.702 (0.645, 0.759)	0.042	0.137 (0.105,0.154)	< 0.001
DL_Post	0.701 (0.647, 0.755)	0.056	0.141 (0.115,0.156)	< 0.001
DL_Longitudinal	0.732 (0.685, 0.779)	0.002	0.128 (0.101,0.143)	< 0.001
Fusion	0.768 (0.731, 0.804)	< 0.001	0.096 (0.075,0.114)	< 0.001
Testing cohort				
Clinical	0.619 (0.511, 0.726)	-	0.183 (0.149,0.201)	-
Delta_radiomics	0.631 (0.525, 0.736)	0.423	0.179 (0.141,0.200)	0.707
DL_Pre	0.656 (0.548, 0.764)	0.287	0.164 (0.124,0.190)	< 0.001
DL_Post	0.643 (0.539, 0.746)	0.348	0.174 (0.129,0.198)	< 0.001
DL_Longitudinal	0.703 (0.599, 0.807)	0.104	0.158 (0.112,0.188)	< 0.001
Fusion	0.734 (0.665, 0.803)	0.021	0.151 (0.120,0.186)	< 0.001

## Data Availability

All data generated or analyzed during this study are available from the corresponding author on reasonable request.

## Abbreviations

ESCC: Esophageal squamous cell carcinoma
CCRT: Concurrent chemoradiotherapy
MRI: magnetic resonance imaging
PET: Positron emission tomography
CECT: Contrast-enhanced CT
OS: Overall survival
IMRT: Intensity-modulated radiation therapy
ROI: Region of interest
GTV: Gross tumor volume
CEL: Cross-scale embedding layer
LSDA: Long short distance attention
Grad-CAM: Gradient-weighted Class Activation Mapping
PCA: Principal component analysis
RSF: Random Survival Forest
iBS: integrated Brier score
CI: Confidence interval
SHAP: Shapley additive explanations
pCR: pathological complete response
