# Supplementary material for: Longitudinal CT-based deep learning radiomics for predicting prognosis in esophageal squamous cell carcinoma treated with definitive chemoradiotherapy: a two-center study
Source: J Cancer. 2026 Mar 30;17(4):808–18. doi: 10.7150/jca.126804 (PMC13105156; doi:10.7150/jca.126804)
Supplement: Supplementary file 1 — Supplementary figures and tables. [file jcav17p0808s1.pdf]

Table. S1 The details of CT image acquisition parameters.

CT scanner	Tianjin Medical University Cancer Institute & Hospital	Xijing hospital
Scanner model	Force	Big Bore CT
Manufacturer	SIEMENS	Phillips
Slice thickness (mm)	2.5 (median)	5 (median)
Field of view (mm^2)	350×350	350×350
Tube voltage (Kv)	120	120
Tube current (mAs)	200-250	200-250
Matrix	512×512	512×512
Contrast agent type	Omnipaque, GE Healthcare, USA	Omnipaque, GE Healthcare, USA
Contrast agent dosage (Infusion)	1.5 ml/kg	1.5 ml/kg
Contrast agent infused rate	3.5 ml/s	3.0 ml/s
Venous phase interval time	60s after injection of contrast agent	70s after injection of contrast agent

Table. S2 Features included in the delta-radiomics model and their coefficients.

	Features	Coef
DRF1	DRF_wavelet.HLL_gldm_DependenceNonUniformity	0.127772386
DRF2	DRF_wavelet.LLH_glcm_DifferenceAverage	-0.114388781
DRF3	DRF_wavelet.HHL_glcm_Contrast	-0.031884803
DRF4	DRF_wavelet.HLL_glszm_LargeAreaEmphasis	-0.066791230
DRF5	DRF_wavelet.HHL_glszm_LowGrayLevelZoneEmphasis	-0.116705968
DRF6	DRF_wavelet.HHL_glcm_Idmn	0.044339414
DRF7	DRF_original_shape_Maximum2DDiameterSlice	-0.024569951
DRF8	DRF_wavelet.LLH_glszm_GrayLevelNonUniformity	0.042772044
DRF9	DRF_wavelet.HHH_glcm_DifferenceEntropy	0.006079389
DRF10	DRF_wavelet.HHH_glcm_ClusterProminence	-0.001259205

Table. S3 Multivariate Cox proportional hazards regression.

Variable	HR	Lower_CI	Upper_CI	P
Age				
<65	ref			
≥65	0.848	0.589	1.221	0.375
Sex				
Male	ref			
Female	1.408	0.744	2.666	0.294
Tumor Location				
Proximal third	ref			
Middle third	0.949	0.623	1.446	0.807
Distal third	0.963	0.567	1.634	0.888
Clinical T Stage				
T1-2	ref			
T3	6.388	0.878	46.479	0.067
T4a	7.007	0.947	51.844	0.057
T4b	7.344	0.971	55.526	0.053
Clinical N Stage				
N0-1	ref			
N2	1.145	0.76	1.726	0.517
N3	1.3	0.752	2.247	0.347
Clinical M Stage				
M0	ref			
M1	1.021	0.583	1.786	0.943
Delta_GTV	0.99	0.669	1.465	0.961
group				
low_risk	ref			
high_risk	7.325	4.568	11.746	<0.001

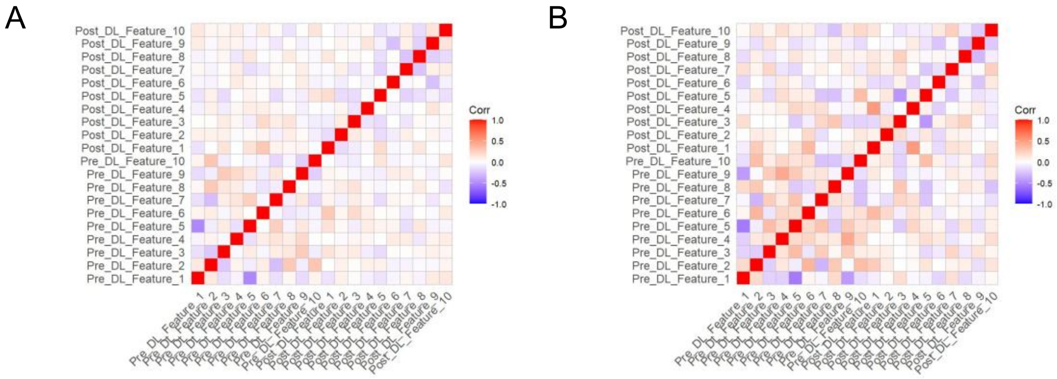

Figure. S1 Correlation coefficients of deep-learning features in the (A) training cohort and (B) testing cohort.

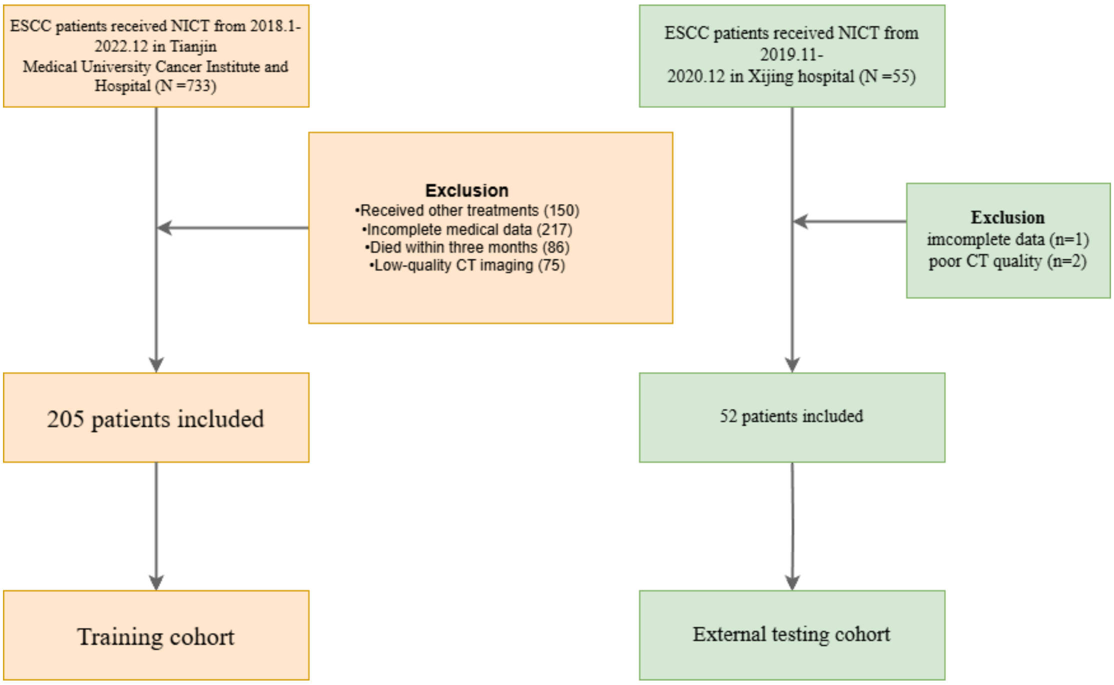

Figure. S2 Flow diagram shows patient selection and exclusion.

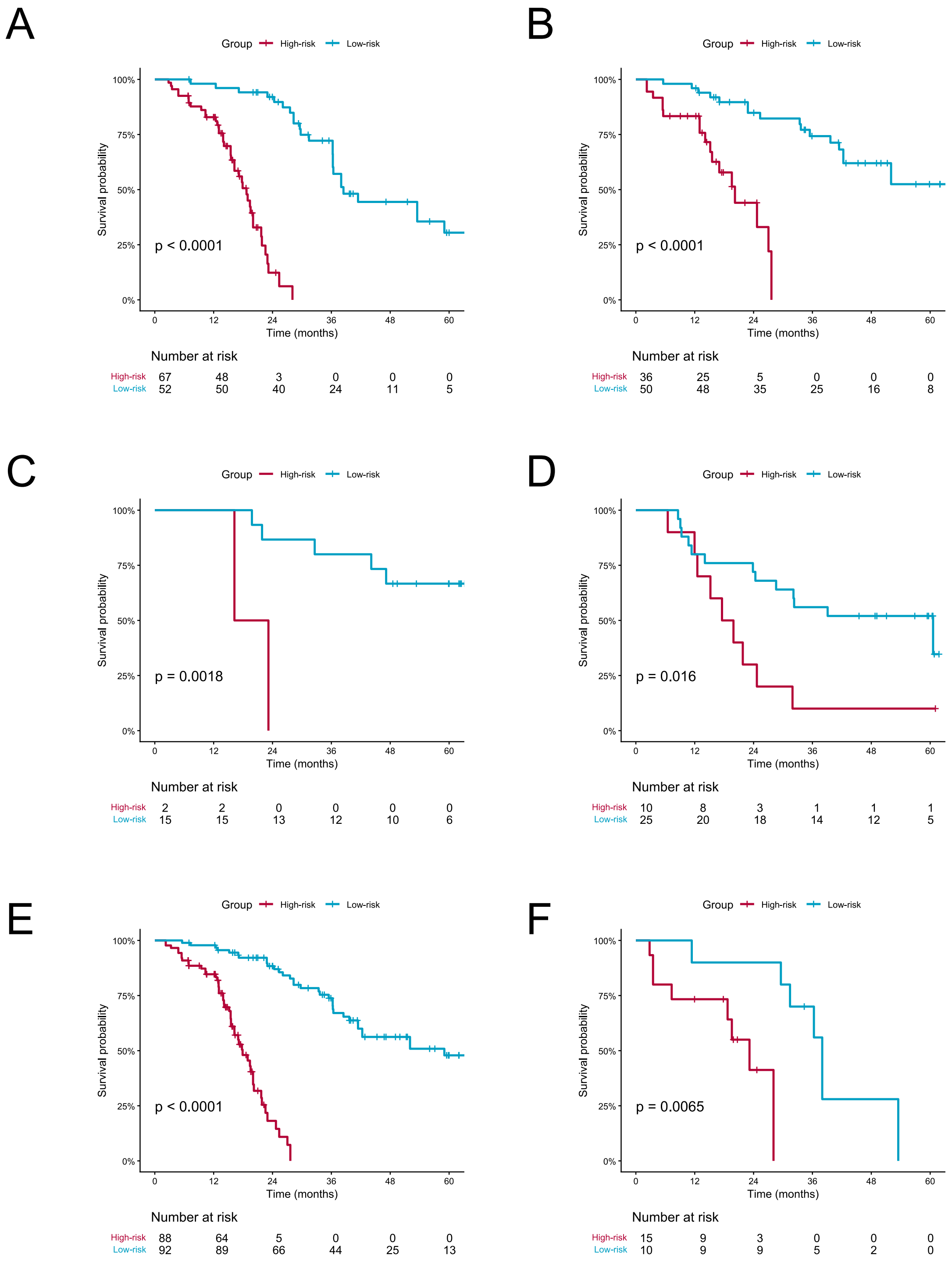

Figure. S3 Subgroup analysis of OS among patients with age<65y and age⩾65y in the (A, B) training and (C, D) testing cohorts. E-F Subgroup analysis of OS among patients with M0 and M1 in the training cohort.
